# Enteral feeding is associated with longer survival in the advanced stages of prion disease

**DOI:** 10.1093/braincomms/fcz012

**Published:** 2019-09-10

**Authors:** Kirsty McNiven, Akin Nihat, Tze How Mok, Selam Tesfamichael, Veronica O’Donnell, Peter Rudge, John Collinge, Simon Mead

**Affiliations:** 1 National Prion Clinic, National Hospital for Neurology and Neurosurgery, University College London Hospitals NHS Foundation Trust, London WC1N 3BG, UK; 2 MRC Prion Unit at UCL, UCL Institute of Prion Diseases, 33 Cleveland Street, London W1W 7FF, UK

**Keywords:** prion, CJD, enteral, RIG, PEG

## Abstract

To report the frequency, complications, survival and motivations for enteral feeding in UK patients with prion diseases. We analysed data from an ongoing prospective observational cohort study of UK patients with prion diseases (*n* = 635). Gastrostomy-treated cases were matched by age, gender, disease aetiology, severity, duration and a genetic predictor of survival (ratio 1:3.1). The main outcome was survival (unadjusted log-rank test); secondary outcomes were future functional impairments, complications and retrospective carer interviews to determine qualitative benefits and motivations. Enteral feeding is uncommon in UK patients with prion diseases (*n *= 26/635; 4.1%), but more frequent in acquired (7/41, 17.1%) and inherited (7/128, 5.5%) compared with sporadic disease (12/466, 2.6%; *P* = 3 × 10^−5^ chi-squared), and used mostly at advanced stages. Enteral feeding was complicated by infection and the need for reinsertions, but associated with markedly longer survival at advanced neurodisability (median 287 days, range 41–3877 versus 17 days, range 0–2356; log-rank test in three aetiologies each *P* < 0.01). Interviews revealed different motivations for enteral feeding, including perceived quality of life benefits. We provide Class II evidence that enteral feeding prolongs the akinetic-mute phase of all aetiological types of prion disease. These data may help support decision making in palliative care. Enteral feeding is an important potential confounder in prion disease clinical trials that use survival as an endpoint.

## Teaser

We analysed data from a prospective observational cohort study of UK patients with prion disease. Using a matched case–control design, we found enteral feeding prolongs the akinetic-mute phase of all types of prion disease, but does not appear to preserve useful functions. Enteral feeding was complicated by infections and reinsertions.

## Graphical Abstract

**Graphical Abstract:**
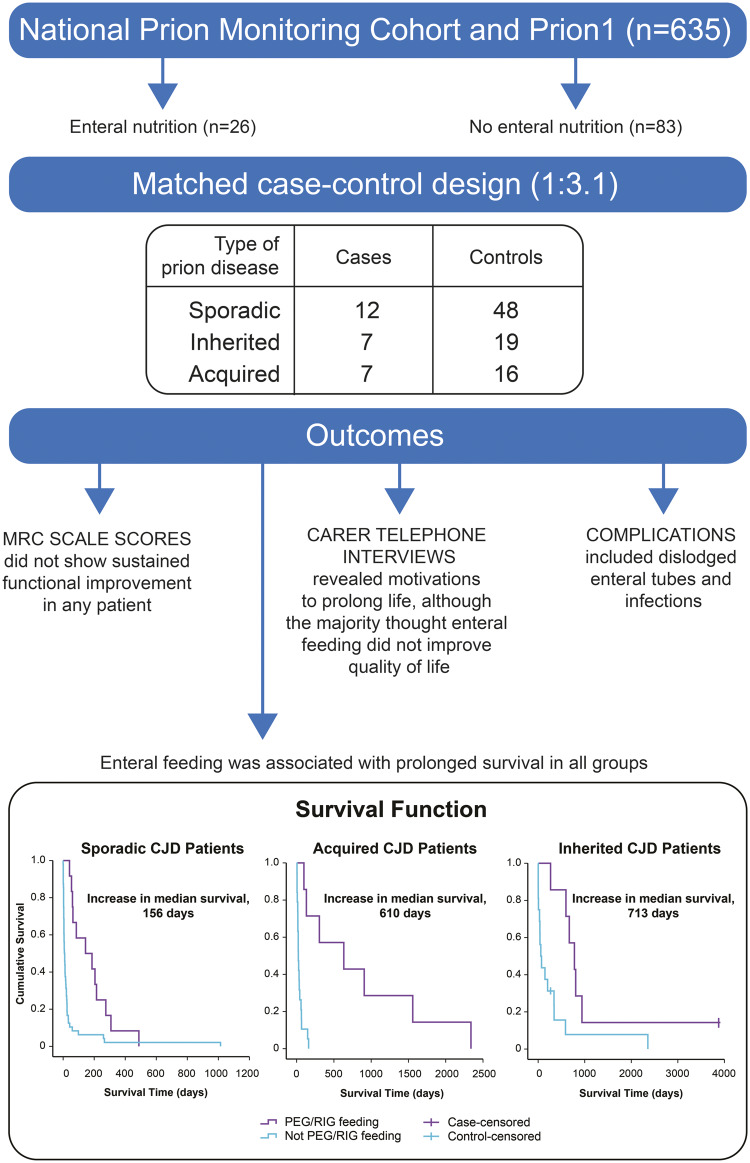

## Introduction

Prion diseases are a group of often rapidly progressive neurodegenerative conditions characterized by the conversion of normal cellular prion protein (PrP) into abnormal, disease-associated forms. The most common type, sporadic Creutzfeldt-Jakob disease (sCJD), typically presents as a rapidly progressive dementia associated with cerebellar ataxia and myoclonus, leading to akinetic-mutism usually in a few months. Less common are genetic forms associated with mutation of the PrP gene and forms acquired through iatrogenic or dietary exposure to prions. Survival in prion disease is strongly influenced by disease aetiology, and in sCJD the genotype at an amino-acid polymorphism at codon 129 of the PrP gene (Palmer *et al.*, 1991; Pocchiari *et al.*, 2004; Mead *et al.*, 2016).

Most, if not all, prion disease patients will develop dysphagia during the course of their illness (Thompson *et al.*, 2013). Evidence of outcomes in amyotrophic lateral sclerosis and other more common forms of neurodegenerative disease are contradictory and practices vary widely (Sampson *et al.*, 2009; EFNS Taskforce on Diagnosis and Management of Amyotrophic Lateral Sclerosis *et al.*, 2012; Stavroulakis *et al.*, 2013; ProGas Study Group, 2015). The use of artificial feeding in patients with dementia poses clinical and ethical dilemmas (Sampson *et al.*, 2009). A large minority of prion disease patients in Japan are managed with enteral feeding, with some evidence of a survival benefit (Iwasaki *et al.*, 2017); however, there is no available Class II evidence, or any evidence in other populations that this management alters survival or quality of life. Clinical trials in prion disease typically use survival as an endpoint, but the degree to which this might be confounded by artificial feeding is unclear.

This study aims to provide evidence to support decision making about enteral feeding in prion disease based on longitudinal prospective data acquired in the UK National Prion Monitoring Cohort study.

## Materials and Methods

### Participants

Patients were recruited to a sub-study of the National Prion Monitoring Cohort Study (incorporating the MRC PRION-1 trial) between 2004 and 2015 (Thompson *et al.*, 2013). In brief, the National Prion Monitoring Cohort is a prospective observational interval-cohort study of patients with probable or definite (autopsy confirmed) prion disease, with frequent face-to-face and telephone follow-up. Diagnosis of probable sCJD or acquired CJD was based on established diagnostic criteria; genetic analysis of the PrP gene was done by sequencing in 95% of cases in the sub-study to diagnose inherited prion disease and determine the genotype at codon 129 of the PrP gene. Demographic characteristics were recorded at enrolment. Treatment with enteral feeding via percutaneous endoscopic gastrostomy (PEG) (*n* = 5) or radiologically inserted gastrostomy (RIG) (*n *= 21) was documented in case report forms; those who had <2 weeks of nasogastric feeding were excluded. In 2013, the National Prion Monitoring Cohort was used to develop a Rasch-modelled 20-point functional composite outcome measure, the Medical Research Council Prion Disease Rating Scale (MRC Scale), recorded in all patients (Thompson *et al.*, 2013). In brief, an MRC Scale score of 20/20 implies independence of all activities of daily living, whereas a score of 2/20 or less implies an akinetic-mute state.

### Sub-study design

We used a matched case–control design. A regression model using the entire National Prion Monitoring Cohort was an alternative design consideration; however, based on recent experience of clinical trial modelling, we were confident that we would not be able to achieve adequate model fit across different aetiological categories (Mead *et al.*, 2016). Each case was matched with up to four controls, by diagnosis, age, gender, codon 129 genotype and disease severity and duration (measured by the MRC Scale). No patients dropped out of the study and all but one case and one control had died at the time of analysis.

### Statistical analysis

Survival was analysed by log-rank test using SPSS v22 (IBM Inc.).

### Telephone interview

Telephone contact was attempted with the family contact for each case in 2017, to assess their experiences of enteral feeding. Closed question responses were quantified, open-ended responses were recorded as handwritten verbatim by the interviewer and responses were read back to the relatives to ensure accuracy. Qualitative responses were collated and explored for common narratives; quotes are used to illustrate each question and represent the perspective of the relative.

### Ethics

Both studies were approved by the Eastern Medical Research Ethics Committee, London and are compliant with the Declaration of Helsinki.

### Data availability

The data that support the findings of this study are available from the corresponding author (S.M.), upon reasonable request.

## Results

### Cohort sub-study

Twenty-six of 635 participants (12 sCJD, 7 acquired CJD and 7 inherited prion disease; *P* = 3 × 10^−5^ chi-squared test with more than expected in acquired and inherited categories) were identified as having enteral feeding through to death. Post-mortem was undertaken and confirmed diagnosis in 14 of 26 cases and 44 of 83 controls; neuropathological features were similar in both groups. The average MRC Scale when enteral feeding commenced was 3.2/20 for sCJD, 6/20 for acquired CJD and 4/20 in inherited CJD (difference not statistically significant by *t*-test). Twenty-three cases and 80 controls were Caucasian, the remaining 3 cases and 3 controls were of other ethnic origin. Three patients made the decision themselves to pursue enteral feeding with the full support of relatives; two presented with dysphagia unusually close to disease onset and the third, with PrP systemic amyloidosis and marked weight loss, required it as a means of additional nutritional support unrelated to dysphagia (Mead *et al.*, 2013)—despite the different indication, this patient commenced enteral feeding at an advanced disease state consistent with other participants.

**Figure 1:**
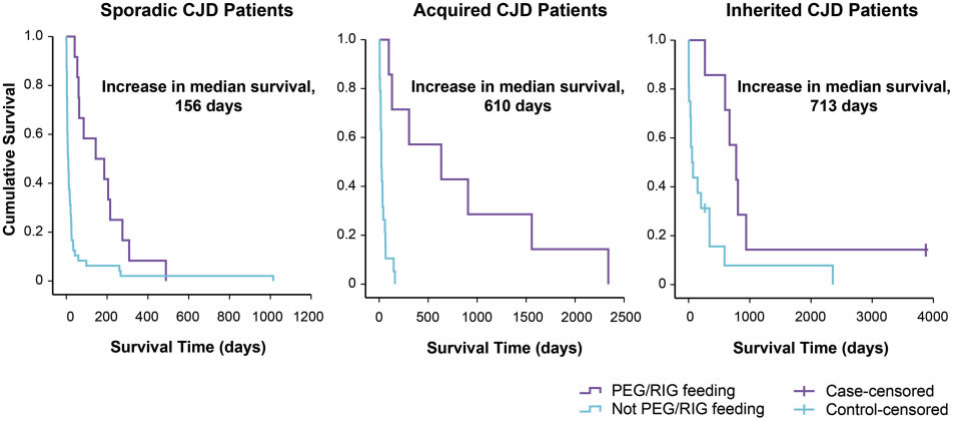
**Survival post-enteral feeding.** Cases are shown in purple, controls in blue. Censored cases and controls were alive at the time of data analysis.

Eighty-three controls were matched (see Table 1, by aetiology *P* = 0.56; gender *P* = 0.86; age *P* = 0.07; MRC Scale *P* = 0.36; genotype at codon 129 *P* = 0.99; mutation of the PrP gene *P* = 0.97; disease duration *P* = 0.22). The main clinical indication for enteral feeding was dysphagia. From the date of percutaneous endoscopic gastrostomy/RIG insertion to death (Fig. 1), patients with sCJD survived a median of 165 days (range 41–488 days) compared with 9 days in controls (range 0–1014 days), *P* < 0.001 log-rank test. Acquired CJD patients survived a median of 633 days (range 98–2339 days) compared with 23 days in controls (range 5–161 days) *P* < 0.001 log-rank test. Inherited CJD survived a median of 778 days (range 267–3877 days) compared with 65 days in controls (range 2–2356 days), *P* < 0.01 log-rank test. There was no difference in survival between those who had nasogastric feeding (*n *= 3) for <2 weeks compared to other controls (84, 112 and 398 days).

**Table 1:** Case-control data in the enteral feeding substudy of the National Prion Monitoring Cohort

		Cohort (excluding asymptomatic & at-risk)	Cases				Controls			
			Sporadic	Acquired	Inherited	All cases	Sporadic	Acquired	Inherited	All controls
Age at onset	Mean	61	62	29	43	48	65	34	51	55
	Median	64	63	23	42	51	65	30	49	58
	Range	13-88	22-77	17-48	25-63	17-77	47-78	17-54	37-69	17-78
Gender	Female	332	4	4	6	14	30	3	10	43
	Male	303	8	3	1	12	18	16	6	40
Enrolment MRC Scale Score	Mean	7	6	13	7	8	6	14	13	9
	Median	6	6	13	8	8	3.5	15	14	9
	Range	0-20	0-18	3-19	2-17	0-19	0-18	3-20	1-19	0-20
Codon 129 genotype	MM	299	4	6	2	12	16	15	8	39
	MV	164	3	1	4	8	12	4	8	24
	VV	106	4	0	1	5	16	0	0	16
	Not Tested	66	1	0	0	1	4	0	0	4
Disease duration (Symptom onset date-DOD, days)	Mean	516	519	1395	2810	1490	264	639	698	639
	Median	207	529	991	1690	862	179	296	260	296
	Range	32-11108	85-874	39-3429	973-9755	39-9755	45-2231	201-709	135-7914	45-7914
First MRC Scale Score post PEG/RIG insertion	Mean		1.8	3.3	1.1		1.8	3.3	5.3	
	Median		2	2	1		1	2	3	
	Range		0-4	1-9	0-5		0-9	0-15	0-14	
Survival post PEG (DOD-PEG/RIG insertion date, days)	Mean		177	853	1133	616	45	41	281	90
	Median		165	633	778	287	9	23	65	17
	Range		41-488	98-2339	267-3877	41-3877	0-1014	5-161	2-2356	0-2356

### Complications and functional outcomes

MRC Scale scores indicated an advanced disease state in the large majority of patients when enteral feeding was started. All but one patient died by the time of analysis. Post-percutaneous endoscopic gastrostomy/RIG MRC Scale scores did not provide evidence of sustained improvement of MRC Scale in any single patient. Several complications were seen: the tubes were dislodged, usually due to patient agitation, at least once in 38% of cases, tube site infections occurred in 23%. Poor skin condition (42%) and chest infections (65%) were seen during follow-up in cases.

### Telephone interviews

Of all 26 family or carer contacts, 13 were available to answer questions about enteral feeding. Reflecting on the motivations to pursue enteral feeding, six relatives wanted to do as much as they could to prolong life; ‘you can’t just give up’ one said. In two cases, outstanding diagnostic uncertainty was a factor as there was hope a treatable alternative diagnosis might be made, ‘not feeding wasn’t an option’. For those patients who chose enteral feeding themselves, relatives considered it improved quality of life. However, the majority thought enteral feeding did not improve quality of life; ‘it prolonged the inevitable’, ‘he was just there’. When asked if enteral feeding met expectations, those who agreed, did so because it ‘extended life’; others who were unsure, stated ‘it made me feel better’, ‘it made my life slightly more bearable, knowing she wasn’t hungry’. Finally, when asked if they would make the same decision to pursue enteral feeding, 8/13 relatives agreed that they would. The remainder, who would not, thought it ‘was very stressful’, ‘was not beneficial’ and ‘prolonged the inevitable’.

## Discussion

Our study compared survival and functional outcomes of prion disease patients with enteral feeding tubes to matched controls as a sub-study of a prospective observational cohort. Similar to a previous report in the Japanese population (Iwasaki *et al.*, 2015), we found that enteral feeding appeared to prolong survival in the akinetic-mute phase of the disease. There were complications of treatment, and there was no sustained improvement in function in any single patient. Motivations for enteral feeding were diverse, with retrospective carer experiences mixed. These data provide evidence to support future decision making about gastrostomy, which we generally discourage. The markedly longer survival in those fed enterally could bias the analysis of survival in clinical trials if tube feeding is associated with treatment allocation.

There are limitations to the matched-control design: power is reduced by selection from the Cohort study, and control matching might have introduced bias. The alternative of using regression analysis of the entire Cohort is problematic in this disease setting as we cannot confidently fit an adequate model across aetiologies and modifier genotypes, which include powerful effects on survival and small samples. We minimized loss of power by selecting up to four controls per case.

A Cochrane review (2009) found insufficient evidence to suggest the benefits of enteral feeding in non-specific dementia over non-enteral feeding in terms of survival, nutrition, functional status, pressure ulcers or infections (Sampson *et al.*, 2009). Studies evaluating enteral feeding in amyotrophic lateral sclerosis are mixed, and recommend that when gastrostomy is being considered, bulbar symptoms, malnutrition, respiratory function and the patient’s general condition should be taken into account (EFNS Taskforce on Diagnosis and Management of Amyotrophic Lateral Sclerosis *et al.*, 2012) as in this condition, substantial weight loss before gastrostomy may adversely affect survival outcome (Stavroulakis *et al.*, 2013; ProGas Study Group, 2015). Our study adds to the heterogeneity of experiences of enteral feeding in neurodegenerative diseases.

The decision to go ahead with enteral feeding is complex and ethically challenging. Evidence may be useful to help inform families/carers in future discussions with their clinicians. Dysphagia commonly presents in the advanced stage in CJD. Our findings that enteral feeding can significantly prolong life in a state of highly advanced neurodisability may be relevant for families considering this difficult dilemma; in particular, given a proportion of relatives/carers who found it not beneficial subjectively, and the experience thereafter distressing.

## Abbreviations

ALS: amyotrophic lateral sclerosis
CJD: Creutzfeldt-Jakob disease
NPMC: National Prion Monitoring Cohort
MRC Scale: Medical Research Council Prion Disease Rating Scale
PEG: percutaneous endoscopic gastrostomy
PrP: prion protein
RIG: radiologically inserted gastrostomy
